# Trends in cervical cancer incidence and survival in Estonia from 1995 to 2014

**DOI:** 10.1186/s12885-018-5006-1

**Published:** 2018-11-07

**Authors:** Kristiina Ojamaa, Kaire Innos, Aleksei Baburin, Hele Everaus, Piret Veerus

**Affiliations:** 1 Oncology Center, East Tallinn Central Hospital, Ravi 18, 10138 Tallinn, Estonia; 2 Department of Epidemiology and Biostatistics, National Institute for Health Development, Hiiu 42, 11619 Tallinn, Estonia; 3 Haematology-Oncology Clinics, Tartu University Hospital, L. Puusepa 1a, 50406 Tartu, Estonia; 4 Estonian Cancer Screening Registry, National Institute for Health Development, Hiiu 42, 11619 Tallinn, Estonia

## Abstract

**Background:**

Cervical cancer (CC) incidence in Estonia is the third highest in Europe, even though an organised nation-wide screening program has been in place since 2006.

The aim of the study was to analyse the incidence and survival of CC in Estonia, focusing on age, morphology and stage at diagnosis.

**Methods:**

Data from Estonian Cancer Registry were used to analyse age-standardized (world) and age-specific incidence for 1968–2014 rates. Joinpoint regression was used to estimate the annual percentage change (APC) for incidence trends. Age-period-cohort model was used to summarise time trends in terms of cohort and period effects. Relative survival ratios (RSR) were calculated for cases diagnosed in 1995–2014. Union for International Cancer Control version 7 of the TNM classification for malignant tumours was used to categorise stage.

**Results:**

The age-standardized incidence of CC increased since 1980s at a rate of 0.8% per year. A significant increase was seen for all age groups except for 70+. The incidence of squamous cell carcinoma mimicked the overall trend, while adenocarcinoma showed increase since mid-1990s (APC 6.7). Age-period-cohort modelling showed strong cohort effects with the lowest risk for birth-cohorts born around 1940 and significantly increasing risks for successive cohorts born thereafter. No period effects were seen.

The proportion of stage IV cases increased from 13% in 2005–2009 to 18% in 2010–2014. A significant increase was seen in the overall 5-year RSR from 1995 to 1999 to 2010–2014 (58% vs 66%). In 2010–2014, the 5-year RSRs ranged from 89% in women aged 15–39 to 41% in age group 70+. For stages I to IV, the respective RSRs were 98, 74, 57 and 22%.

**Conclusions:**

The inadequate uptake and insufficient quality of the Pap-smear based screening program has not brought along a decline in the incidence of CC in Estonia. Stage distribution has shifted towards later stages. New approaches are needed to prevent CC in Estonia.

## Background

Cervical cancer (CC) is the second most common gynaecological cancer in Estonia [1]. In 2014, 177 new cases were diagnosed. According to Volume XI of Cancer Incidence in Five Continents, Estonia had the third highest incidence of CC among European countries in 2008–2012: the age-standardized (world) incidence rate in Estonia was 18 per 100,000, which was comparable to many developing countries [2]. The age-standardized mortality in 2013 (4.3 per 100,000) ranked ninth in Europe [3]. From EUROCARE-3 (1990–1994) to EUROCARE-5 (2000–2007), the European average 5-year relative survival estimates for CC did not change (62% for both periods) and neither did the highest observed estimates (around 70%). The relative survival rate for Estonian CC patients, however, increased from 53 to 64% [4, 5].

CC is a highly preventable cancer. The role of Human papilloma virus (HPV) as its main risk factor is well-established [6] and the natural development of CC from premalignant lesions to invasive cancer can be detected and treated [7]. A decline in the incidence and mortality of CC has been seen in many developed countries [8–10], mainly in accordance with effective population-based screening programs that have resulted in decreasing incidence and mortality rates and a shift towards diagnosis at earlier stages [11, 12]. In Estonia, however, increasing incidence and stable mortality have been demonstrated in previous studies [9, 10], despite the nation-wide organized screening program in place since 2006.

The aim of the study was to analyse the incidence and relative survival of CC in Estonia, with particular focus on age, birth-cohorts, morphology and tumour stage.

## Methods

For this register-based study, data on incident cases of invasive CC (ICD-O-3 codes C53.0; C53.1; C53.8; C53.9) were obtained from the Estonian Cancer Registry (ECR), a population-based registry that covers the whole country with a population of 1.32 million in 2014 and has data since 1968. Population denominator data were obtained from Statistics Estonia. Age-standardized (world standard population [2]) and age-specific incidence trends were analysed for 1968–2014. Age-standardised incidence was also analysed by morphological subtypes, classified based on ICD-O-3: squamous cell carcinoma (SCC), adenocarcinoma (AC), not otherwise specified (NOS), and other. Joinpoint analysis with Joinpoint Regression Program (version 4.1.1.1) from the Surveillance Research Program of the US National Cancer Institute (http:// surveillance.cancer.gov/joinpoint/) was used to model the rates and calculate the estimated annual percent change (APC) with 95% confidence intervals (95% CI). In addition, we analysed CC incidence trends with age-period-cohort analysis as shown by Carstensen [13]. In order to avoid age-period-cohort identification problem, arbitrary constraints were added to the model. We chose 1935–1939 as the reference cohort. To evaluate the goodness of fit between models with different parametrization, we used Akaike Information Criterion [14]. The age-function is shown as age-specific rates in the reference cohort adjusted for the period effect. The cohort function is expressed as a rate ratio relative to the reference cohort and the period function as a rate ratio relative to the age–cohort prediction [13].

For the age-period-cohort analysis, incidence cases were aggregated into 5-year period and age groups. Due to small numbers the cases younger than 30 years and older than 79 years were excluded. Thus, the analysis included 10 age groups (30–34…75–79), 9 periods (1970–1974…2010–2014) and 27 birth-cohorts (1895–1899…1980–1984). For calculations we used Epi package’s apc.fit function in R software (http://www.R-project.org).

For survival analysis, we used data on all adult (≥15 years) cases of invasive CC diagnosed in Estonia during 1995–2014, regardless of cancer sequence. The patients were followed up for vital status until December 31, 2014 via linkage with the Estonian Population Registry; in case of death or emigration, the respective date was ascertained. The linkage was done using unique personal identification numbers. Death certificate only (DCO) cases and those diagnosed at autopsy were excluded from survival analyses.

Age at diagnosis was categorized into six groups: 15–29; 30–39; 40–49; 50–59; 60–69 and 70+ years. Pathological or clinical TNM stage was reported by clinicians and/or pathologists on notification forms and this information was available for cases diagnosed since 2005. Summary stage was categorised according to the Union for International Cancer Control version 7 of the TNM classification: stage I (T1(1a-1b2)N0 M0); stage II (T2(2a-2b)N0 M0); stage III (T3/3aN0M0; T3bNanyM0; T1–3N1M0); stage IV (T4NanyM0; TanyNanyM1). Microinvasive and invasive carcinomas were included. Non-invasive cases categorised as carcinoma in situ (Tis) were excluded. Category “unknown” included cases with no or incomplete information on TNM stage.

Relative survival ratios (RSR) were calculated as the ratio of the observed survival of CC patients to the expected survival of the underlying general population. The latter estimate was calculated according to the Ederer II method [15] using national life tables for female population stratified by the single year of age and calendar year. Cohort analysis was used to estimate one-year and five-year RSRs for patients diagnosed during 1995–1999, 2000–2004, and 2005–2009. Period analysis was used for the latest period 2010–2014. The International Cancer Survival Standards were used for age-standardizing overall RSRs [16]. All calculations were conducted with STATA 14.1 (StataCorp LP, College Stations TX USA); survival analysis was performed using the *strs* module.

The study protocol was approved by the Tallinn Medical Research Ethics Committee.

## Results

**Fig. 1:**
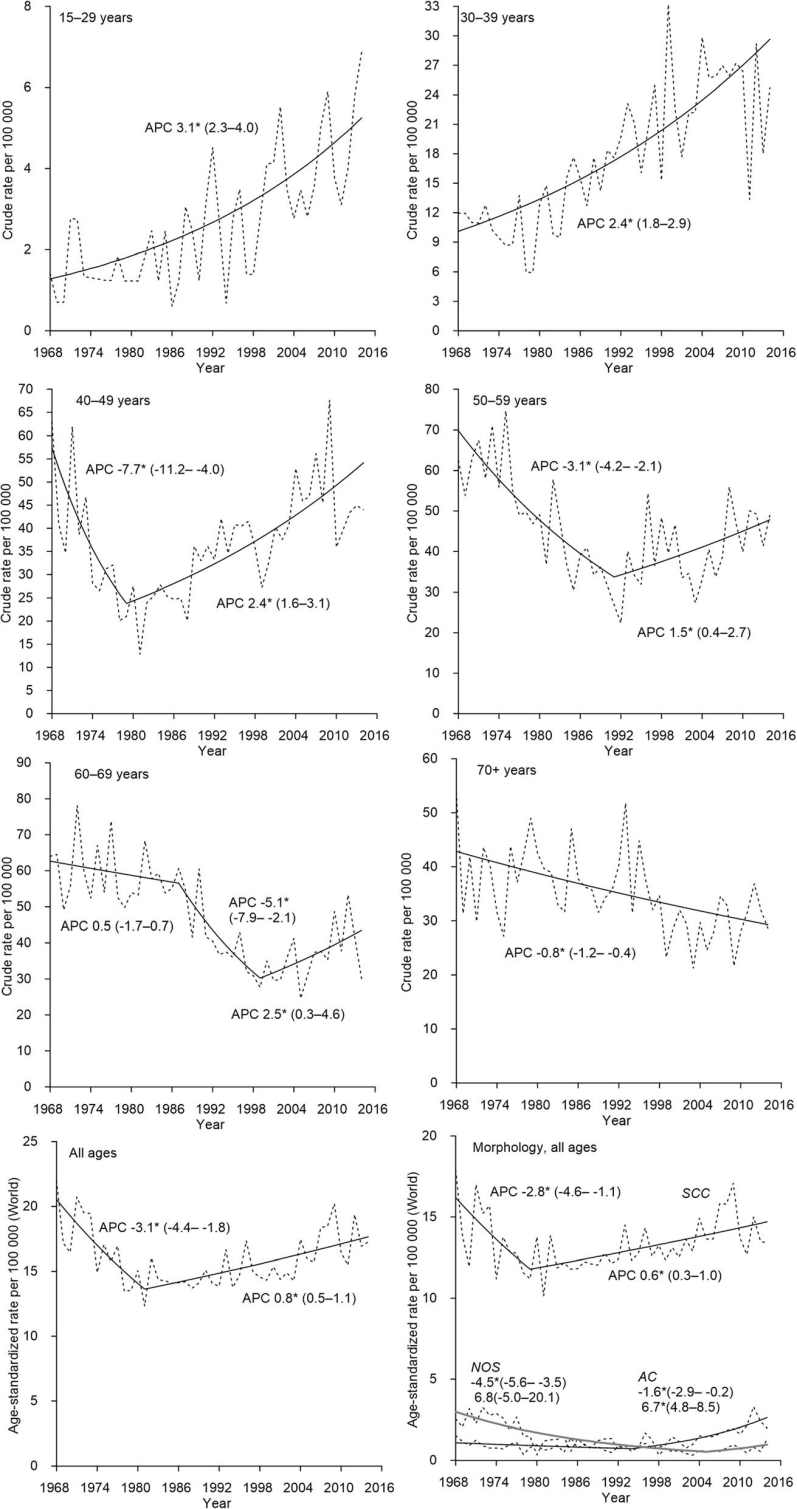
Observed (dotted line) and modelled (solid line) rates and annual percentage change (APC) for trends in incidence (1968–2014) of cervical cancer in Estonia. *The APC is significantly different from zero at alpha = 0.05

The age-standardized incidence of CC in Estonia declined from 1968 to 1980 (APC 3.1) and started to increase thereafter at a rate of 0.8% per year (Fig. 1). In the two youngest age-groups (15–29 and 30–39), a continuous and significant increase was seen over the entire study period, while in all age groups between 40 and 69 years, a significant decline was seen until 1980s or 1990s, followed by a significant increase. For women aged 70 years and older, the incidence decreased over the entire study period at a rate of 0.8% per year.

**Fig. 2:**
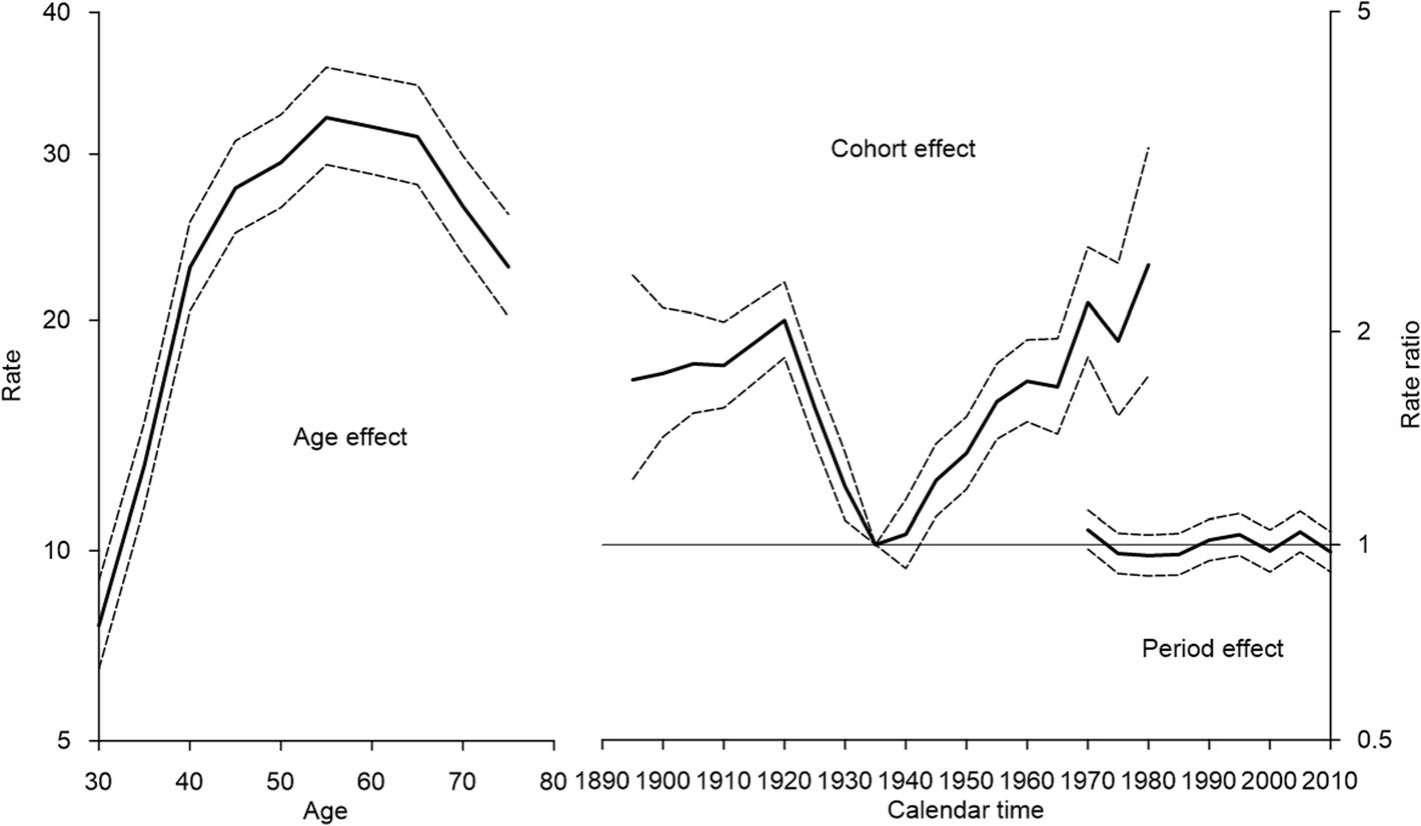
Estimated age-period-cohort effects for incidence of cervical cancer in Estonia, 1970–2014. On the x-axis, 5-year age groups, birth-cohorts and calendar periods are defined by the first year of the interval. Reference cohort is 1935–1939

The age-period-cohort analysis showed decreasing incidence rate ratios for women born between 1920 and 1940 and constantly increasing risk for women born after 1940s (Fig. 2). No period effects were seen in CC incidence. The peak incidence occurred in ages 50–60 years and then slowly declined with age.

The incidence trend of SCC, the main histological subtype of CC, mimicked the overall trend and increased at a rate of 0.6% per year starting from 1979 (Fig. 1). In AC incidence, a significant increase of 6.7% per year was seen over the past twenty years.

**Fig. 3:**
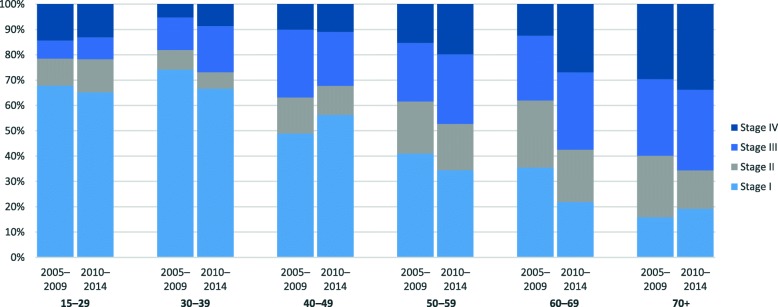
TNM stage distribution of cervical cancer cases diagnosed in Estonia, 2005–2014 (cases with unknown stage excluded)

After the exclusion of 21 DCO cases and 13 autopsy cases, 3403 cases were available for survival analysis (Table 1). The overall percentage of microscopically verified cases was 98% and this quality indicator remained stable over the study period. Overall, women were most frequently diagnosed at the age of 40–49 years, followed by age group 50–59 years. The proportion of AC increased, and the proportion of SCC decreased. Overall, TNM stage distribution shifted significantly towards later stage at diagnosis from 2005 to 2009 to 2010–2014, as the proportion of stage I cases decreased from 40 to 35% (*p* = 0.0096) and the proportion of stage IV cases increased from 13 to 18% (*p* = 0.0019). The proportion of stage I cases was 59% in age-group 15–29, 67% in age-group 30–39, and decreased with age in older age groups (15% in age group 70+) (Fig. 3). The proportion of cases diagnosed in stage I decreased in nearly all age groups.

**Table 1:** Incident cases of cervical cancer in Estonia, 1995–2014

	Total		1995–1999		2000–2004		2005–2009		2010–2014		p-value^a^
	No	%	No	%	No	%	No	%	No	%	
Total^b^	3403	100	812	100	796	100	900	100	895	100	
Microscopically verified	3327	97.8	791	97.4	782	98.2	882	98.0	872	97.4	*p* = 0.578
Age at diagnosis											
15–29	104	3.1	17	2.1	29	3.6	29	3.2	29	3.2	*p* = 0.008
30–39	439	12.9	109	13.4	108	13.6	122	13.6	100	11.2	
40–49	840	24.7	192	23.6	210	26.4	251	27.9	187	20.9	
50–59	780	22.9	189	23.3	158	19.8	210	23.3	223	24.9	
60–69	612	17.9	152	18.7	153	19.2	135	15.0	172	19.2	
70+	628	18.4	153	18.8	138	17.3	153	17.0	184	20.6	
Morphology											
AC	327	9.6	52	6.4	66	8.3	87	9.7	122	13.6	*p* < 0.001
SCC	2845	83.6	690	85.0	688	86.4	763	84.8	704	78.7	
NOS	167	4.9	52	6.4	30	3.8	39	4.3	46	5.1	
Other	64	1.9	18	2.2	12	1.5	11	1.2	23	2.6	
Stage^c^											
I	673	37.5					364	40.4	309	34.5	*p* = 0.002
II	271	15.1					149	16.6	122	13.6	
III	405	22.6					196	21.8	209	23.4	
IV	281	15.7					117	13.0	164	18.3	
Unknown	165	9.2					74	8.2	91	10.2	

^a^chi-square test

^b^death certificate only cases (*n* = 21) and autopsy cases (*n* = 13) excluded

^c^TNM stage information available only from 2005

The age-standardised 5-year relative survival increased significantly from 58% in 1995–1999 to 66% in 2010–2014 (Table 2). An increase was seen in all age-groups except the oldest, and the change was significant for women aged 30–39 and 40–49 years. The latest 5-year RSRs ranged from 89% in women aged 15–39 to 41% in age group 70+. The difference between the 5-year RSR for SCC and AC increased slightly, as a significant rise of 10 percentage units was seen for SCC. A small increase in the 5-year RSRs was observed for stages I–III from 2005 to 2009 to 2010–2014.

**Table 2:** Relative survival ratios (RSR) of cervical cancer in Estonia by age, morphology and stage, 1995–2014

	1995–1999			2000–2004			2005–2009			2010–2014^a^		
	1-year RSR	5-year RSR	95%CI	1-year RSR	5-year RSR	95%CI	1-year RSR	5-year RSR	95%CI	1-year RSR	5-year RSR	95%CI
Total	80	59	55–62	84	64	60–68	86	69	66–72	84	**67**	**64–70**
Total (age-standardized)	78	58	54–62	83	62	58–66	84	67	63–70	83	**66**	**62–69**
Age at diagnosis												
15–29	94	77	49–91	93	83	64–93	86	80	60–90	97	89	70–97
30–39	85	67	57–75	95	82	73–88	98	91	84–95	96	**89**	**82–94**
40–49	84	63	55–69	88	71	64–77	91	76	71–81	92	**80**	**74–85**
50–59	85	59	52–66	86	58	49–65	88	67	59–73	83	61	54–67
60–69	78	60	51–68	81	58	50–67	88	66	57–74	84	66	57–73
70+	64	43	33–53	70	48	37–58	61	42	33–52	67	41	32–50
Morphology												
AC	79	55	40–69	88	66	52–78	85	65	54–75	86	63	52–72
SCC	83	61	57–65	86	65	61–69	88	72	68–75	87	**71**	**68–75**
NOS	51	42	27–55	52	33	17–52	50	38	22–54	41	30	17–44
Stage^b^												
I							100	96	92–98	100	98	95–100
II							96	72	63–80	95	74	65–82
III							81	52	44–59	88	57	49–64
IV							47	24	16–33	53	22	15–29
Unknown							66	49	37–61	65	43	33–53

^a^statistically significant change in 5-year RSR from first to last period marked in bold

^b^TNM stage information available only from 2005

## Discussion

The study showed a steady long-term increase in the age-standardized incidence of CC in Estonia, starting from early 1980s. The overall incidence rates reflected the increasing risk in successive birth-cohorts born after 1940s, with no period effects to counteract them. The improvement of relative survival was more pronounced in younger women. A nation-wide organized CC screening program was started in Estonia in 2006. Screening is provided to women aged 30 to 55 years with valid health insurance. A conventional Pap-smear is taken every 5 years after a negative screening result. The attendance rate of screening program has remained as low as 35% of the target population, while an estimated 80% of women have done Pap-smears outside of the screening program [8, 17].

After a considerable decline in the beginning of the study period, the age-standardized incidence has been steadily increasing in Estonia since early 1980s. Similar trends have been observed in other Eastern European countries [18]. In our study, the age-specific incidence rates increased in all age-groups except in women aged 70+ years. This finding is consistent with the decreasing risk among women born between the two World Wars and the increasing risk in successive birth-cohorts born after the 1940s, also seen in other European countries [9]. However, in the Nordic countries and other high-income countries in Europe, screening has intervened with this risk increase, resulting in a definite decline in CC incidence shortly after the introduction of screening [9]. In Canada, the incidence peaks in birth-cohorts born in 1950 and later were two times lower than in those born before 1920 and it was explained by the high screening participation rate (up to 80%) [19].

An Italian study analysed the results of a newly established organised population-based screening program and showed an increase in early CC incidence right after the start of the program and subsequently, a 25% decrease in age-standardized CC incidence 8 years later [11]. In Bulgaria the opportunistic screening has resulted in plateauing of CC incidence rates [20]. No period-specific screening effects were apparent in other Eastern-European countries [9]. Our results suggest non-effective screening activities in Estonia even though program-based screening is ongoing and opportunistic screening is widely performed. In Eastern European countries, the changes in the health care system after regaining independence and the lack of resources for prevention and healthcare have been suggested as possible causes for the current epidemiological situation with CC [21]. Lower mean income, distance from the nearest well-equipped hospital and the level of education have been established as adverse factors for invasive CC on a more personal level [22, 23].

Our data showed increasing incidence among very young women aged 15–29 who are not targeted by screening in Estonia or elsewhere. It has been found that screening does not have a protective role against CC mortality in that age group [24]. Therefore, more attention should be paid to the prevention of CC among very young women. Vaccination against HPV common cancerous subtypes is highly effective in preventing CC [25]. From 2018, HPV vaccination for girls aged 12–14 was added to the national immunization program in Estonia.

Age-specific analyses showed a peak incidence rate around the age of 55 and declining rates in older age groups. A Nordic study demonstrated that the decline in the incidence of elderly women and the peak incidence at the age of 50 could be the result of an unscreened population [26]. This is caused by the natural history of CC that reaches clinically detectable and invasive features by the age of 45–60 years [27]. In Canada, the long lasting widespread screening resulted in a decline in the incidence and mortality in all age-groups from 1970s to early 2000s, but the largest changes were seen in ages 45 years and older, with reductions as high as 74% in mortality and 69% in incidence [19].

Besides the incidence increase of SCC subtype that mimicked the overall trend, a rapid rise in the incidence of AC subtype was observed from mid-1990s. Such trend was expected based on the results of previous population-based studies [28, 29], as cytology screening is often inefficient in preventing AC. However, it has been shown that screening helps to detect AC at an earlier stage [30]. Premalignant lesions of SCC subtype are clinically more detectable and therefore, the incidence of invasive SCC of the cervix has declined in many countries [31, 32].

We observed an overall decrease in the proportion of stage I cancers and an increase in the proportion of stage IV cancers, which was not expected based on data from previous studies [11, 20]. Data on TNM stage was not available for the pre-screening period. In Bulgaria, the most common stage at diagnosis of CC changed from stage II to stage I; stage I accounted for 35% and stage IV only for 4.5% of new diagnoses over the period of 1993–2013 [23]. In our study, the proportion of cases diagnosed at stage I decreased from 40 to 35% from 2005 to 2009 to 2010–2014 (in comparison, the latter estimate was 60% in Norway), while the latest proportion of stage IV cases was 18% in Estonia and only half of that (9%) in Norway [33]. The observed stage shift in Estonia may be partly explained by the increasing availability of better diagnostics resulting in more accurate staging. Nevertheless, the latest stage distribution is clearly unfavourable. Low participation in screening, the absence of a screening registry and no regular quality assurance within the organised cervical cancer screening programme until 2015 are the most likely reasons for the observed stage distribution.

The overall 5-year relative survival of CC in Estonia improved over the study period and reached 66% in 2010–2014. Data from the Nordic countries for the same period showed the highest 5-year RSR of 72% in Norway [34]. In the Nordic countries, women aged below 50 years had the 5-year RSRs as high as 86–92% (84% in Estonia, data not shown) [34]. Large survival deficit for Estonian patients was seen in age-group 50–59 years (61% in Estonia, 68–75% in the Nordic countries) [34]. The proportion of stage I among this age-group in Estonia was only 31% in the latest period, even though these women should have been screened at least once prior to the diagnosis. This might explain the observed lower survival. For older age-groups, the Estonian estimates were similar to the Nordic data [34] and comparable to other population-based studies [28, 35]. A large age gap of 48 percentage units was seen in 5-year relative survival. A similar gradient was seen in EUROCARE-5 analysis [5]. It has been recognized that older age is associated with more advanced stages [35, 36], also confirmed by our data. In this age group, treatment choices are limited and should be more individualized due to intolerable toxicities and comorbidities. A Japanese study concluded that comorbidities and older age are not as strong predictive survival factors as the missing screening test during earlier life that would have been helped to detect premalignant lesions or cancer at earlier stage [37].

Besides screening as the cornerstone of survival improvement, advanced surgical techniques, modern radiotherapy and the availability of chemotherapy have the potential for improving survival for more advanced stages. The relative survival of CC patients in Estonia is similar to survival in countries with high quality cancer care. By stage, the Estonian survival estimates were quite comparable with Norwegian [33] or Irish data [38]. It should be taken into account, however, that in countries with effective screening programs, the cases that have developed into invasive CC may be more aggressive and therefore have a poorer prognosis.

The main strength of the study was the use of data from a population-based cancer registry that covers the whole country, and the availability of TNM stage and morphology data. The study has several limitations. Possible misclassification of CC cases as “unspecified” should be considered. However, the average annual number of incident cancer cases coded as “Uterus, NOS” (ICD-O-3 C55.9) or “Female genital tract, NOS” (C57.9) in the ECR was only two over the study period. The ECR does not have information whether a case was screen-detected or not. Estonian screening registry started work only in 2015 and therefore no linkage was possible to obtain additional information for the study period. Also, the quality management of screening program and the quality of screening tests were not evaluated at the time. The socioeconomic status of women, as well as individual participation in screening or visits to a gynaecologist, were unknown.

## Conclusions

The study showed several differences in the incidence and survival for CC in Estonia in comparison with other European countries with well-established screening programs. Similarities could be seen with Eastern European countries, suggesting the ineffectiveness of PAP-smear based screening in the region.

The study showed increasing CC incidence in all age-groups except the oldest. These trends were consistent with the increasing risk in successive birth-cohorts born after 1940s. Unfortunately, no period effects were apparent to suggest the effect of opportunistic or organised nation-wide screening. Screening reduces the burden of CC by two mechanisms – reducing incidence through detection and treatment of premalignant lesions and detecting malignant tumours at an earlier stage while still susceptible to curative treatment, − neither of which has been effective in Estonia. Both overall and stage-specific survival of CC in Estonia has improved and is comparable to other European countries.

A stronger input from the government to establish an effective screening program is urgently needed. Both the organisation of screening to augment the continuous monitoring and the quality control of screening activities need to be improved. Women without health insurance as the highest risk group for CC, should be included in the national screening program. The effect of screening should be further studied by taking into account the personal history of Pap-smears and participation in organised screening. The vaccination of girls has just started in 2018, but a change from cytology based to HPV-based cervical screening should be strongly considered.

## Abbreviations

AC: adenocarcinoma
APC: annual percent change
CC: cervical cancer
DCO: death certificate only
ECR: Estonian Cancer Registry
HPV: human papilloma virus
RSR: relative survival ratio
SCC: squamous cell carcinoma

## References

[1] Estonian Cancer Registry. National Institute for Health Development. Available at http://tai.ee/en/r-and-d/registers/estonian-cancer-registry. Accessed 11 Nov 2017.

[2] Bray F, Colombet M, Mery L, et al. Cancer incidence in five continents. Vol. XI (electronic version). Lyon: International Agency for Research on Cancer Available from: http://ci5.iarc.fr. Accessed 11 Nov 2017.

[3] World Health Organization, Department of Information, Evidence and Research, mortality database. Available at http://apps.who.int/healthinfo/statistics/mortality/whodpms/. Accessed 08 Oct 2017.

[4] Sant M, Aareleid T, Berrino F (2003). EUROCARE-3: survival of cancer patients diagnosed 1990-94 – results and commentary. Ann Oncol.

[5] Sant M, Lopez M, Agresti R (2015). Survival of women with cancers of breast and genital organs in Europe 1999-2007: results of the EUROCARE-5 study. Eur J Cancer.

[6] Bosch F, Lorincz A, Munoz N (2002). The causal relation between human papillomavirus and cervical cancer. J Clin Pathol.

[7] Schiffman M, Castle P, Jeronima J (2007). Human papilloma virus and cervical cancer. Lancet.

[8] Elfström K, Arnheim-Dahlström L, Karsa L (2015). Cervical cancer screening in Europe: quality assurance and organisation of programmes. Eur J Cancer.

[9] Vaccarella S, Lortet-Tieulent J, Plummer M (2013). Worldwide trends in cervical cancer incidence: impact of screening against changes in disease risk factors. Eur J Cancer.

[10] Arbyn M, Raifu A, Weiderpass E (2009). Trends of cervical cancer mortality in the member states of the European Union. Eur J Cancer.

[11] Serraino D, Gini A, Taborelli M (2015). Changes in cervical cancer incidence following the introduction of organized screening in Italy. Prev Med.

[12] Landy R, Pesola F, Castanon A (2016). Impact of cervical screening on cervical cancer mortality: estimation using stage-specific results from a nested case-control study. Br J Cancer.

[13] Carstensen B (2007). Age-period-cohort models for the Lexis diagram. Stat Med.

[14] Akaike H (1974). A new look at the statistical model identification. IEEE Trans Autom Control.

[15] Ederer F, Heise H (1959). Instructions to IBM 650 programmers in processing survival computations. Methodological note no. 10.

[16] Corazziari I, Quinn M, Capocaccia R (2004). Standard cancer patient population for age standardising survival ratios. Eur J Cancer.

[17] Anttila A, Ronco G, Working group on the registration and monitoring of cervical Cancer screening Programmes in the European Union; within the European network for information on Cancer (EUNICE) (2009). Description of the national situation of cervical cancer screening in the member states of the European Union. Eur J Cancer.

[18] Vaccarella S, Franceschi S, Zaridze D (2016). Preventable fractions of cervical cancer via effective screening in six Baltic, central, and eastern European countries 2017-2040: a population-based study. Lancet Oncol.

[19] Dickenson J, Stankiewicz A, Popadiuk C (2012). Reduced cervical cancer incidence and mortality in Canada: national data from 1932 to 2006. BMC Public Health.

[20] Samson K, Haynatzki G, Soliman A (2016). Temporal changes in the cervical cancer burden in Bulgaria: implications for eastern european countries going through transition. Cancer Epidemiol.

[21] Maver P, Seme K, Korac T (2013). Cervical cancer screening practices in central and eastern Europe in 2012. Acta Dermatovenerologica.

[22] Leinonen M, Campbell S, Klungsoyr O (2017). Personal and provider level factors influence participation to cervical cancer screening: a retrospective register-based study of 1.3 million women in Norway. Prev Med.

[23] Ulinskas K, Aleknaviciene B, Smailyte G (2013). Demographic differences in cervical cancer survival in Lithuania. Cent Eur J Med.

[24] Vicus D, Sutradhar R, Lu Y (2014). The association between cervical cancer screening and mortality from cervical cancer: a population-based case-control study. Gynecol Oncol.

[25] Baldur-Felskov B, Dehlendorff C, Junge J (2014). Incidence of cervical lesions in Danish women before and after implementation of a national HPV vaccination program. Cancer Causes Control.

[26] Lynge E, Lönnberg S, Törnberg S (2017). Cervical cancer incidence in elderly women-biology or screening history?. Eur J Cancer.

[27] Gustafsson L, Adami H (1989). Natural history of cervical neoplasia: consistent results obtained by an identification technique. Br J Cancer.

[28] Loren L, Bertaut A, Hudry D (2015). About invasive cervical cancer: a French population based study between 1998 and 2010. Eur J Obs Gyn Repr Biol.

[29] Bray E, Carstensen B, Møller H (2005). Incidence trends of adenocarcinoma of the cervix in 13 European countries. Cancer Epidemiol Biomark Prev.

[30] Castanon A, Landy R, Sasieni P (2016). Is cervical screening preventing adenocarcinoma and adenosquamous carcinoma of the cervix?. Int J Cancer.

[31] Wang S, Sherman M, Hildesheim A (2004). Cervical adenocarcinoma and squamous cell carcinoma incidence trends among white women and black women in the United States for 1976-2000. Cancer.

[32] Schiffman H, Wentzensen N (2013). Human papillomavirus infection and the multistage carcinogenesis of cervical Cancer. Cancer Epidemiol Biomark Prev.

[33] Cancer Registry of Norway (2015). Cancer in Norway 2014-Cancer incidence, mortality, survival and prevalence in Norway.

[34] Engholm G, Ferlay J, Christensen N, Kejs, et al. NORDCAN: Cancer incidence, mortality, prevalence and survival in the Nordic countries, version 7.3 (08.07.2016). Association of the Nordic Cancer registries. Danish Cancer Society Available from http://www.ancr.nu. Accessed 15 Sept 2017.

[35] Wright J, Chen L, Tergas A (2015). Population –level trends in relative survival for cervical cancer. Am J Obstet Gynecol.

[36] Klint Å, Tryggvadóttir L, Bray F (2010). Trends in the survival of patients diagnosed with cancer in female genital organs in the Nordic countries 1964-2003 followed up the end of 2006. Acta Oncol.

[37] Ioka A, Tsukuma H, Ajiki W (2015). Influence of age on cervical Cancer survival in Japan. Jpn J Clin Oncol.

[38] National Cancer Registry Ireland. Cancer trends 35 – Cervical cancer. Available at: https://www.ncri.ie/data/survival-statistics. Accessed 06 Apr 2018.

